# Genome Analysis of *Endotrypanum* and *Porcisia* spp., Closest Phylogenetic Relatives of *Leishmania*, Highlights the Role of Amastins in Shaping Pathogenicity

**DOI:** 10.3390/genes12030444

**Published:** 2021-03-20

**Authors:** Amanda T. S. Albanaz, Evgeny S. Gerasimov, Jeffrey J. Shaw, Jovana Sádlová, Julius Lukeš, Petr Volf, Fred R. Opperdoes, Alexei Y. Kostygov, Anzhelika Butenko, Vyacheslav Yurchenko

**Affiliations:** 1 Life Science Research Centre, Faculty of Science, University of Ostrava, 71000 Ostrava, Czech Republic; amandatabitaalbanaz@gmail.com (A.T.S.A.); kostygov@gmail.com (A.Y.K.); 2 Faculty of Biology, M. V. Lomonosov Moscow State University, 119991 Moscow, Russia; jalgard@gmail.com; 3 Biomedical Institute, São Paulo University, São Paulo 05508, Brazil; jayusp@hotmail.com; 4 Department of Parasitology, Faculty of Science, Charles University, 12844 Prague, Czech Republic; JovanaS@seznam.cz (J.S.); volf@cesnet.cz (P.V.); 5 Institute of Parasitology, Biology Centre, Czech Academy of Sciences, 37005 České Budějovice, Czech Republic; jula@paru.cas.cz; 6 Faculty of Science, University of South Bohemia, 37005 České Budějovice, Czech Republic; 7 de Duve Institute, Université Catholique de Louvain, 1200 Brussels, Belgium; Fred.Opperdoes@uclouvain.be; 8 Zoological Institute of the Russian Academy of Sciences, 199034 St. Petersburg, Russia; 9 Martsinovsky Institute of Medical Parasitology, Tropical and Vector Borne Diseases, Sechenov University, 119435 Moscow, Russia

**Keywords:** leishmaniinae, genome analysis, gene gain, gene loss

## Abstract

While numerous genomes of *Leishmania* spp. have been sequenced and analyzed, an understanding of the evolutionary history of these organisms remains limited due to the unavailability of the sequence data for their closest known relatives, *Endotrypanum* and *Porcisia* spp., infecting sloths and porcupines. We have sequenced and analyzed genomes of three members of this clade in order to fill this gap. Their comparative analyses revealed only minute differences from *Leishmania* *major* genome in terms of metabolic capacities. We also documented that the number of genes under positive selection on the *Endotrypanum*/*Porcisia* branch is rather small, with the flagellum-related group of genes being over-represented. Most significantly, the analysis of gene family evolution revealed a substantially reduced repertoire of surface proteins, such as amastins and biopterin transporters BT1 in the *Endotrypanum/Porcisia* species when compared to amastigote-dwelling *Leishmania*. This reduction was especially pronounced for δ-amastins, a subfamily of cell surface proteins crucial in the propagation of *Leishmania* amastigotes inside vertebrate macrophages and, apparently, dispensable for *Endotrypanum/Porcisia*, which do not infect such cells.

## 1. Introduction

Trypanosomatids (family Trypanosomatidae) is a diverse group of mono-flagellated kinetoplastids, which unites obligate parasites of invertebrates (monoxenous species, one-host developmental cycle) with those, shuttling between invertebrates and vertebrates or plants (dixenous species, two-host developmental cycle) [1,2]. The following five genera represent the latter group—*Trypanosoma*, *Leishmania*, *Phytomonas*, *Porcisia*, and *Endotrypanum.* Dixenous trypanosomatids evolved from monoxenous ones independently at least three times [3]. One such transition had happened within the subfamily Leishmaniinae [4,5], giving rise to the prominent genus *Leishmania*. Biology of its representatives has been extensively studied due to its medical importance, leading to the well-resolved taxonomy of this genus and its closest relatives [6,7]. The genus *Leishmania* is currently subdivided into four subgenera—*Leishmania*, *Mundinia*, *Sauroleishmania*, and *Viannia*, which are well-defined based on their biology (host or vector specificity and clinical manifestations) and phylogeny [8]. Many of these parasites have been scrutinized using modern genomic methods and the comparative analyses have revealed their relationships and evolutionary history [9,10,11,12,13]. At the same time, the closest phylogenetic relatives of *Leishmania*, specifically the genera *Endotrypanum* and *Porcisia*, remained neglected and did not attract much of attention for the reasons that are discussed below.

Mesnil and Brimont described an enigmatic intra-erythrocytic flagellate in 1908 from a French Guianan two-toed sloth (*Choloepus didactylus*) and named *Endotrypanum schaudinni* [14]. Its intracellular localization was subsequently confirmed using electron microscopy [15]. This species turned out to be very unusual, as the intra-erythrocytic forms were represented by epimastigotes, while, in culture, only promastigotes, reminiscent of *Leishmania* spp., could be observed [16]. This led to a suggestion that the two morphotypes belong to distinct lineages, of which the intra-erythrocytic parasite represents an unidentified trypanosome, while the cultured forms are related to *Leishmania* [17]. It is currently accepted that both of the morphotypes belong *to Endotrypanum* [8,18]. Of note, *E. colombiensis* [19] and *E. herreri* [20] parasitize different white cells in sloth blood. They also produce amastigotes in tissue culture, but, unlike *Leishmania* spp., those of *E. colombiensis* die out [19]. Neither species can infect hamsters *in vitro*, but *E. colombiensis* was associated with cutaneous and visceral leishmaniases in men and dogs in Colombia and Venezuela [21,22].

Besides *Endotrypanum*, two other somewhat mysterious parasites, named *Leishmania hertigi* and *L. deanei*, were described from the American tropical porcupines [23,24]. Biochemical and molecular studies have shown that these flagellates are related, yet distinct from *Leishmania* spp. [17,25], which leads to the erection of a new genus *Porcisia* to accommodate them [8]. Another name, *Paraleishmania*, was proposed for this taxon [4], but it did not become formally available according to the article 16.1 of the International Code of Zoological Nomenclature. Although only two species of this genus have been described so far; it is conceivable that others will be discovered in the future, given that there are 17 porcupine species present in the Americas. Both flagellate species can be found in the upper dermis of the skin, and in the liver and spleen of their vertebrate hosts [23,26,27]. They cause no apparent pathology, except for the vacuolization of the host cell’s cytoplasm. Some of the flagellates even appear to be extracellular [24]. In culture, these parasites proliferate as long aciculate nectomonad-like promastigotes, morphologically resembling those of *L.* (*Mundinia*) spp. [28]. No lesions were observed in experimental infections of hamsters using intradermal inoculation of culture. Parasites from the inoculation site could be introduced into culture for up to a year, although amastigotes could only be microscopically detected for a few weeks [23]. The absence of any pathology in the natural host and their long-term survival in experimental animals indicates that these parasites have developed sophisticated mechanisms of evading the host’s immune system, which are probably responsible for their high infection rates seen in natural populations. 

There is good evidence from the experimental [29] and natural [30] infections that *Endotrypanum* spp. use phlebotomine sand flies as vectors and develop in their hindgut and pylorus, as do *L.* (*Viannia*) spp. This makes it difficult to distinguish the two groups of parasites in the wild sand flies. However, unlike *L.* (*Viannia*) spp., *Endotrypanum* can also be detected in Malpighian tubules [31,32]. *Endotrypanum schaudinni* has been documented in six Brazilian sand fly species [30], whereas *E. colombiensis* and *E. equatorensis* only appear to be vectored by the Panamanian and Ecuadorian *Lutzomyia hartmanni* [19,33]. Overall, there is evidence of vector specificity, with infection rates varying between species. For example, the rate of *E. schaudinni* infection of *Lu. gomezi* was significantly higher than that of *Lu. sanguinaria*, which suggests that the former is a more susceptible natural vector [34]. So far, there are no clear leads as to the transmission of *Porcisia,* but a recent study has identified *Lu. antunesi* as a potential vector for *P. hertigi* [35]. 

According to phylogenetic inferences, the *Endotrypanum*-*Porcisia* clade separated from *Leishmania* 70–120 MYA, in the Cretaceous period [36,37], when placental mammals (that emerged ~66 MYA, after the Cretaceous-Paleogene boundary) did not yet exist [38]. Xenarthrans, one of the most ancient groups of placental mammals in South America, are hosts for *Endotrypanum* spp. It seems plausible that the parasite clade under study, to which *Endotrypanum* belongs, has originated in this mammalian lineage. However, *Porcisia* spp. have switched to other suitable hosts, including the ancestral American porcupines (Erethizontidae).

In this work, we sequenced the genomes of three species of the *Endotrypanum—Porcisia* clade and performed their comparative analyses, demonstrating correlations between their genomic content and biological peculiarities.

## 2. Materials and Methods

### 2.1. Cultivation, DNA Isolation and Species Verification

The strains that were studied in this work were *Porcisia deanei* TCC258 (MCOE/BR/91/M13451), which were isolated from *Coendou* sp. in Brazil in 1991, *P. hertigi* TCC260 (MCOE/PA/80/C8), isolated from *Coendou rothschildi* in Panama in 1980, and *Endotrypanum* sp. ATCC 30507 (MCHO/PA/72/3130) isolated from the sloth’s (*Choloepus* sp.) blood in Panama in 1972 and representing the *E. monterogeii* group B in [39]. Promastigotes were cultivated in M199 medium (Sigma−Aldrich, St. Louis, MO, USA) supplemented with 10% heat-inactivated fetal bovine calf serum (Thermo Fisher Scientific, Waltham, MA, USA), 1% Basal Medium Eagle vitamins (Sigma−Aldrich, St. Louis, MO, USA), 2% sterile urine, and 250 μg/mL of amikacin (Bristol-Myers Squibb, New York, NY, USA). The total genomic DNA was isolated from 10 mL of trypanosomatid cultures with the DNeasy Blood & Tissue Kit (Qiagen, Hilden, Germany) according to the manufacturer’s instructions. 18S rRNA and gGAPDH genes were amplified while using primers S762 and S763 [40] and M200 and M201 [41], respectively, following the previously described protocol [42]. PCR amplicons were directly sequenced at Macrogen Europe (Amsterdam, The Netherlands) using internal primers, as described previously [43,44]. The obtained nucleotide sequences were deposited to GenBank under the accession numbers MT862138–MT862140 (18S rRNA) and MT887294–MT887296 (gGAPDH). BLAST analysis confirmed the identity of species under study [45].

### 2.2. Whole-Genome and Transcriptome Sequencing and Annotation

The whole genomes and transcriptomes of *Endotrypanum monterogeii* ATCC 30507, *Porcisia deanei* TCC258 and *P. hertigi* TCC260 were sequenced, as described previously [9] while using the Illumina HiSeq platform. On average, 63 and 46 million 100 nt long paired-end raw reads were produced for genomes and transcriptomes, respectively. The raw reads were trimmed using Trimmomatic v.0.39 [46] with the following settings: ILLUMINACLIP:TruSeq3-PE-2.fa:2:20:10 LEADING:3 TRAILING:3 SLIDINGWINDOW:4:15 MINLEN:75. The read quality before and after the trimming was checked with FastQC v.0.11.8 [47].

The trimmed genomic reads were assembled *de novo* using SPAdes Genome assembler v.3.13.0 [48] with default settings and automatic k-mer selection (k-mers of lengths 21, 33, and 55 nt were used). The resulting scaffolds were checked for potential contamination with BlobTools v.1.1 [49] and those shorter than 500 nucleotides or showing high-quality BLAST hits at the nucleotide level (identity > 95% and coverage > 85%) to sequences outside Euglenozoa in NCBI database were discarded. Using these criteria, 1008 (348,597 bp), 756 (255,773 bp), and 1987 (673,383 bp) sequences for *E. monterogeii*, *P. deanei* and *P. hertigi*, respectively, were identified as contamination (Figure S1). The quality of the resulting assemblies was assessed using QUAST v.5.0.2 [50]. The genome and transcriptome read mapping was performed with Bowtie2 v.2.3.5.1 using “--end-to-end” and “--very-sensitive” options [51] and HISAT2 v.2.1.0 with “--dta-cufflinks” option [52], respectively. The raw reads and assembled genome sequences were deposited to NCBI database under BioProject accession numbers PRJNA680236, PRJNA680237, and PRJNA680239 for *E. monterogeii* ATCC 30507, *P. deanei* TCC258, and *P. hertigi* TCC260, respectively.

Genome annotation using transcriptome evidence was performed in the web-based program Companion with default options [53], using *L. major* Friedlin as the most closely related available reference. The pseudo-chromosome level sequences produced with Companion software were only used for the purpose of synteny analysis, in all other cases scaffold-level sequences produced by Spades assembler were analyzed. The genome completeness and annotation quality were assessed with BUSCO v.3 using the eukaryota_odb9 reference database [54].

### 2.3. Repeats Identification and Synteny Analysis

The *de novo* repeat identification was performed using RepeatModeler v.2.0.1 and RepeatMasker v.4.1.0 [55] with the option ‘-species’ set to Euglenozoa. The repeats families were annotated using BLASTX and BLASTN with the *e*-value set to 0.01.

Synteny analysis was completed using SyMAP v.5.0.5 [56] with the following settings: minimum size of sequence to load, 500 bp; minimum number of anchors required to define a synteny block, 7; synteny blocks merged in case of overlaps; and, only the larger block kept if two synteny blocks overlapped on a chromosome. For synteny inferences, the pseudo-chromosomes that were produced by Companion were used with the sequences of *L. major* Friedlin as a reference. The cross-mapping of pseudo-chromosomes was visualized using Chromosomer v.0.1.4 [57].

### 2.4. Genome Coverage Analysis and Ploidy Estimation

The trimmed genomic reads were mapped onto the genome assembly with Bowtie2 [51] using “-end-to-end” and “-very-sensitive” options. The GenomeCov tool from the BEDTools v.2.28.0–33 package [58] was used to calculate the per-base read coverage for the 50 longest scaffolds. The median genome coverage (represented by the 50 longest scaffolds) was calculated using the dplyr package in R v. 3.6.3 [59]. For ploidy estimation, the relative coverage values were obtained by dividing the average coverage of each of the 50 longest scaffold sequences by the average genome coverage. The ploidy was inferred assuming that the majority of the chromosomes are diploid. The coverage plots were visualized using the R v. 3.6.3 packages ggplot2 [60] and tidyverse [61]. WeeSAM v.1.5 [62] was used to obtain the multiple genome coverage statistics that are represented in Table S2.

### 2.5. Variant Calling

After the genomic reads were mapped, as described above, and, prior to variant calling, the read duplicates were removed and the reads were locally realigned using the MarkedDuplicates and IndelRealigner tools of GenomeAnalysisTK v.4.1.4.0 [63] with default settings, except for REMOVE_DUPLICATES = true. The variant calling was performed using Platypus v.0.8.1 [64] with default settings.

### 2.6. Orthology and Phylogenomic Analyses

The OrthoFinder v.2.3.8 [65] with default settings was used on a dataset of 44 trypanosomatid species with the eubodonid *Bodo saltans* representing an outgroup in order to infer protein orthology. Out of a total 14,511 orthologous groups (OGs), 522 contained proteins that were encoded by single-copy genes. Out of these, 410 OGs with the average percent identity within the group ≥60% were selected for phylogenomic inferences. The amino acid sequences in each OG were aligned using the L-INS-i algorithm in MAFFT v.7.453 with default settings [66] and trimmed using TrimAl v.1.4 [67] with “-strict”, “-sident”, “-sgc”, and “-sgt” options, and then concatenated. The average protein identity within OGs was assessed using the esl-alistat script v.0.46 from HMMER package [68].

The maximum likelihood phylogenetic tree was inferred in IQ-TREE v.1.6.12 [69] with JTT + F + I + G4 being automatically selected as the best fit model and branch support estimated using 1000 standard bootstrap replicates. For the Bayesian inference, two independent chains were run in PhyloBayes-MPI [70] for ~16,000 iterations under the JTT + CAT + G model with the removal of invariant sites. The absolute topological convergence was achieved after 300 iterations. For all run parameters at the end of the analysis, the relative differences were below 0.1 and effective sample sizes ≥596. The final tree was visualized using the dplyr, ggplot2, and ggtree packages in R v. 3.6.3 [71]. 

The gene family gains, losses, expansions, and contractions were analyzed with Dollo’s and Wagner’s (gain penalty set to 3) parsimony algorithms implemented in the COUNT software [72], as described previously [9]. KEGG and Interpro IDs were assigned to the annotated proteins with BlastKOALA [73] and locally installed InterproScan v.5.45-80 with “-dp”, “-goterms”, and “-pathways” settings [74], respectively. OG intersections were inferred and visualized with UpSetR package in R v. 3.6.3 [75]. 

Metabolic pathways were analyzed using “all against all” BLASTP searches with an *e*-value cut-off of 1e^−50^, as described previously [76]. This rather strict *e*-value was chosen in order to distinguish between true orthologous proteins and more distant homologues, which are not necessarily functional orthologues.

### 2.7. Gene Ontology Analysis and Functional Annotation

Gene ontology (GO) identifiers and related GO terms were assigned to the annotated proteins using the InterproScan v. 5.45-80 and QuickGo web server, respectively [77]. When possible, *L. major* proteins were used as representative sequences in these analyses.

### 2.8. Analyses of Amastin Surface Proteins and Biopterin Transporters

Taking the large amastin protein family size in *Leishmania* spp. into account, we restricted our analyses to a subset of ten selected trypanosomatid species/strains: *Crithidia fasciculata*, *E. monterogeii* ATCC30507 and LV88, *Leishmania braziliensis*, *Leishmania major*, *Leptomonas pyrrhocoris*, *P. deanei, P. hertigi, Trypanosoma brucei brucei,* and *Trypanosoma cruzi*. For studying the biopterin BT1 protein family, the whole protein set of 44 trypanosomatids and the outgroup *B. saltans* was used.

We performed the HMM search (HMMER v.3.3.1, [68]) using the amastin (PF07344) and BT1 (PF03092) HMM profiles from Pfam database [78], along with the respective datasets described above. Only hits with *e*-values below 1e^−10^ were kept for further steps. The pairwise identity of the hits was assessed using Clustal Omega 2.1 [79]. For amastins, only the sequences having more than 20% identity to the α-amastin LmjF.28.1400 of *L. major* Friedlin were kept. Of note, in this filtering step, the proto-*δ*-amastin LmjF.34.0970 was formally excluded and, therefore, it is not present on the tree. The same criteria were used to filter BT1 sequences, with the protein identity of the hits being compared to the BT1 of *L. major* Friedlin (LmjF.35.5150). Finally, for both amastins and BT1 transporters, the remaining hits were aligned using the L-INS-i algorithm in MAFFT v.7.453 [66] and only sequences with <90% of gaps were kept. These sequences were then re-aligned and trimmed with TrimAl v.1.4 [67] using the option “-gappyout”. In total, we identified 239 amastin sequences in our dataset and, after applying the filtering criteria mentioned above, 188 sequences were retained and used in phylogenetic analysis. For putative BT1 transporters, 320 sequences were retained out of 544 initial hits.

The ML trees were built using IQ-TREE v.1.6.12 [69], with 1000 bootstrap replicates, and the best-fit models for amastins and BT1 being WAG + F + G4 and JTT + F + I + G4, respectively. The trees were visualized in FigTree v.1.4.4 [80]. For predicting transmembrane domains (TMD), the protein sequences that were presented in the trees were submitted to the TMHMM Server v. 2.0 [81] with the default settings.

We performed a reconstruction of the sequence similarity-based protein network in order to gain some insight into affiliation of the amastins excluded from the phylogenetic analysis according to the filtering criteria mentioned above. In the case of phylogenetic analysis after application of the abovementioned thresholds, 10 amastins out of 15 were retained for *E. monterogeii*, seven out of 10 for *P. deanei*, and five out of eight for *P. hertigi*. The amastin protein network was inferred from a dataset of 237 protein sequences longer than 100 amino acids using EFI-EST [82] with a BLAST *e*-value threshold of 1e^−10^ and a minimum alignment score (roughly corresponding to sequence similarity) set to 30. The result was visualized in Cytoscape v.3.8.0 [83]. In this analysis, only two short sequences were discarded from the original dataset containing 239 HMMER hits, being identified with an *e*-value lower than 1e^−10^. Putative annotations were assigned to the inferred protein clusters based on the results of phylogenetic analysis. Sequences, which were excluded from the phylogenetic analysis by filtering criteria, were annotated based on previously published results [84,85].

### 2.9. Selection Analysis

A subset of six species that includes three investigated *Endotrypanum/Porcisia* spp., *Leishmania major*, *L. tarentolae*, and *Leptomonas seymouri* was used for positive selection analysis. From all OGs, we only selected those that contained sequences of all six species. Tuples of orthologous protein sequences were aligned with MAFFT v.7.453 and multiple alignments were converted into codon alignments using a custom Python script. In order to identify genes under positive selection, a branch-site model A [86] was used for *Endotrypanum/Porcisia* and *Leishmania* branches (two independent tests), while other branches were set as a background. The LRT was used to evaluate whether branch-site model A had a significantly better fit for the codon site with ω > 1 in comparison with the branch-site model A1, which fixes ω to 1.0 on the branches of interest. The analysis was carried out using the ETE3 framework [87]. If positive selection was detected within an OG, a gene of *L. major* was used as a representative sequence for the group. Genes that were under positive selection on the *Endotrypanum/Porcisia* and *Leishmania* branches were subjected to GO enrichment analysis in the top.GO R package [88].

## 3. Results

### 3.1. Endotrypanum Sp. ATCC 30507 (MCHO/PA/72/3130) Is E. monterogeii

Confirming previous results (Table S2 in [39]), 18S rRNA sequence analysis established the identity of *Endotrypanum* sp. ATCC 30507 (MCHO/PA/72/3130) as bona fide *E. monterogeii*. This name is used hereafter. 

### 3.2. General Features of Endotrypanum and Porcisia Genomes

The three genome assemblies that were obtained herein (Figure S1) had total length and N50 values (given in parentheses) of 30.4 Mb (57 kb) for *E. monterogeii*, 29.5 Mb (30.35 kb) for *P. deanei*, and 29.1 Mb (29.55 kb) for *P. hertigi* (Table S1). They were conspicuously shorter than the reference ~32 Mb genome of *L. major* Friedlin. This can be explained by multiple factors, including differences in real gene content and assembly procedures (different scaffolding methods may result in discordant gap content, some repeats may not completely resolved in the absence of long reads, etc.).

The genomes that were sequenced here were predicted to encode about 7600 proteins on average, which is significantly less than in *L. major* Friedlin (8519), but correlates well with the estimated genome sizes of these species (Table S1). The percentages of missing benchmarking universal single-copy and duplicated orthologs (BUSCOs), which are used to estimate completeness of the assembly and annotation quality, are as low as ~20% and 5% for each of the sequenced genomes, similarly to the respective estimates for the high-quality reference genome of *L. major* Friedlin (19.8% and 6.3%, respectively). Along with the results of the coverage homogeneity analysis (described below), this suggests that most of the repeated regions were properly resolved. A very low proportion of homozygous single nucleotide polymorphisms (SNPs) (around 1%) indicates a minimal number of genome assembly errors (Table S1). The variant calling procedure led to the identification of the highest total SNP number (74,038) in the genome of *P. deanei*, while those of *P. hertigi* and *E. monterogeii* displayed less variation with 58,586 and 40,923 SNPs, respectively (Table S1).

### 3.3. Genome Coverage Analysis, Ploidy Estimation and Synteny Analysis

For the analysis of genome assembly coverage and ploidy estimation, genomic reads were mapped back onto the scaffolds (see Materials and Methods). The coverage is uniform across all three analyzed genome assemblies, with the median numbers being 152, 108, and 112 for *E. monterogeii*, *P. deanei*, and *P. hertigi*, respectively (Figure S2). The per-scaffold average proportion of low-coverage sites (the percentage of sites with coverage ≤0.2 of the average depth) is small for all three genome assemblies: 1.67% (*E. monterogeii*), 2.42% (*P. deanei*), and 2.82% (*P. hertigi*) (Figure S2, Table S2). In all three species, most of the scaffolds (~96%) have homogeneous coverage with the coefficient of coverage variation below 1 (Table S2). Most of the 50 longest scaffolds in the obtained assemblies are diploid (2n), and just a few have other ploidy levels (3–4n) (Figure S3, Table S2). The highest rate of aneuploidy was detected in *E. monterogeii*, with five out of 50 largest scaffolds demonstrating estimated ploidy over 2n (Figure S3).

We documented variable levels of gene order conservation among analyzed trypanosomatid genomes, with 41 to 85% of genes located within synteny blocks in the various intra- and interspecies comparisons (Figure S4, Table S3). These numbers are similar to the estimates for other Leishmaniinae, and they are consistent with the majority of trypanosomatid genes located within relatively well-conserved polycistronic transcription units [9,89,90].

### 3.4. Analysis of Repetitive Sequences

Twenty seven families of repeats spanning 3.66% (~1.1 Mb) of the genome assembly were identified in the *E. monterogeii* genome. Out of these, 0.3% are low complexity repeats. *Porcisia deanei* and *P. hertigi* have 40 and 45 families of repeats, covering 4.22% and 4.49% of their genomes (Table S4), with 0.52% and 0.58% of low complexity repeats, respectively. Even though *L. major* Friedlin has a higher number of identified repeat families (321), the genomic spanning of these repeats is also comparable 3.66%, from which 0.38% are low complexity repeats. For most of the identified repetitive sequences (including species-specific groups of repeats), no functional annotation could be inferred (Table S4). Among the annotated families of repetitive sequences, the majority contain surface antigens (leishmanolysin GP63 and protease GP46, GP stands for a glycoprotein), as well as serine/threonine-protein phosphatases, which possibly play a role in cell division and the modulation of host immune response [91,92,93,94,95]. 

### 3.5. Gene Family Sharing Analysis

Annotated proteins of 44 trypanosomatids and *B. saltans* (Table S5) cluster into 14,511 orthogroups (OGs) that contain at least two sequences. OG sharing analysis (group composition is presented in Table S6) shows that 1650 OGs (11.4% of the total OG number), incorporating mostly housekeeping genes, are shared among all kinetoplastid groups in our dataset (Figure S5, Table S7). The analysis of OGs that are uniquely shared among various representatives of the *Endotrypanum*/*Porcisia* clade revealed several dozen of OGs incorporating proteins, to which function could not be assigned with confidence (Table S7). The same analysis performed only for the representatives of the *Endotrypanum*/*Porcisia* clade led to the identification of a large set of 6764 OGs that were shared by all four species/strains (Figure S5, Table S7), which is in agreement with the high synteny levels for genomes of these species. 

### 3.6. Phylogenomic Analysis

**Figure 1:**
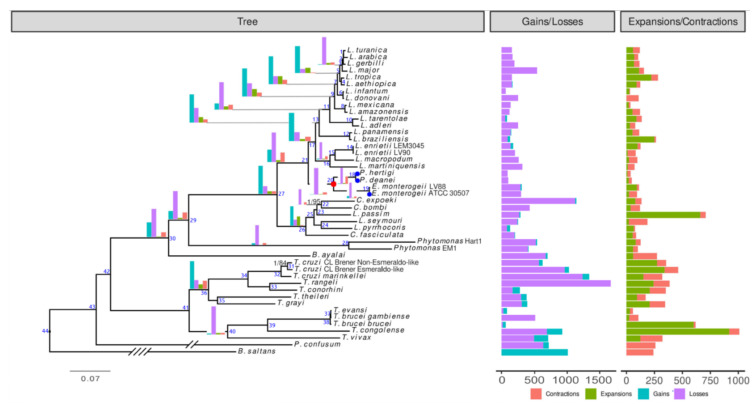
Phylogenomic tree based on 410 proteins encoded by single-copy genes from 44 trypanosomatids and the eubodonid *Bodo saltans,* Posterior probabilities and bootstrap supports are shown (in black) only if the latter is <100%. The scale bar represents substitutions per site. The numbers of orthologous groups (OG) gained/lost/expanded/contracted at certain nodes and leaves (species) are depicted using bar plots placed at the nodes and on the right of the tree, respectively (see Table S8 for exact counts; node numbers indicated in blue correspond to those in the Table S8). The *Endotrypanum*/*Porcisia* node (node 20) and the isolates sequenced in this study are marked with red and blue circles, respectively. The length of *B. saltans* and *P. confusum* branches was reduced four- and two-fold, respectively, for visualization purposes.

The maximum likelihood and Bayesian phylogenomic trees inferred using the supermatrix of 410 proteins encoded by single-copy genes have the same topology and demonstrate maximal supports for almost all branches (Figure 1). This topology is compatible with those inferred previously [2,9,96,97], which confirms the position of the genera *Endotrypanum* and *Porcisia* as the closest known relatives of the genus *Leishmania*. 

### 3.7. Evolution of Gene Families

Aiming at elucidating gene content differences between *Endotrypanum*/*Porcisia* and other trypanosomatids, we performed a genome-wide analysis of gene content with the emphasis on genes and genes families gained/lost/expanded/contracted at the *Endotrypanum*/*Porcisia* branch (node 20 in the Figure 1), revealing evolutionary changes on this branch as compared to other Leishmaniinae. In addition, we systematically examined the differences in metabolic pathways between *Endotrypanum*/*Porcisia* and *L. major* (below). The *Endotrypanum*/*Porcisia* node is characterized by the prevalence of gene family losses and contractions over gains and expansions (node 20 in the Figure 1, Tables S8–S10), 150 and seven-fold, respectively. This is reminiscent of the situation that was inferred for the subgenus *Leishmania* (*Mundinia*) [9]. No functional annotation could be confidently assigned to the OGs gained and expanded at the ancestral *Endotrypanum*/*Porcisia* node (Tables S9 and S10). Among OGs lost and contracted at this node, ~70% and 37% of families, respectively, are represented by hypothetical proteins. We also analyzed the genus-specific changes in the gene family repertoire, focusing on the evolutionary events at the *Porcisia* and *Endotrypanum* nodes (Figure 1, nodes 18 and 19, respectively). Similar to the ancestral *Endotrypanum*/*Porcisia* node, both of the nodes are dominated by gene family losses, with the majority of proteins having no functional annotation. Several protein families at this node have undergone noticeable evolutionary changes in their composition and size, including membrane proteins (transporters, cell surface proteins), proteins involved in cell signaling (kinases, phosphatases, GTPases, adenylate cyclase-like proteins), subtilisins and peptidases, families of housekeeping genes encoding motor proteins (actin, dynein, myosin, and kinesin), as well as ribosomal and DNA repair proteins (Table S9). Out of these, we analyzed, in detail, amastins and biopterin transporter family BT1, the two protein families displaying the most significant changes and, at the same time, playing a key role in host-parasite interactions and triggering the host immune system response [98]. Changes in the repertoire of these proteins may represent an adaptive mechanism for the successful evasion of the host immune system and be associated with lower pathogenicity. 

### 3.8. Amastins

Amastins are a large family of transmembrane glycoproteins (GPs) that are widely conserved across trypanosomatids and expressed mainly during the amastigote stage of their life cycle [84,85,99]. These GPs are among the most immunogenic surface antigens in *Leishmania*, enabling parasites to invade host cells and provide other advantages, such as fast and efficient response to the changes of physiological conditions inside macrophages [100]. The number of genes encoding putative amastins vary across *Leishmania* spp., with the highest counts being documented for the representatives of *Leishmania* and *Viannia* subgenera, such as *L. infantum* (68 proteins), *L. major* (63 proteins), and *L. braziliensis* (66 proteins) (Tables S11 and S12). In the representatives of the *Endotrypanum/Porcisia* clade, there are from 8 (*P. hertigi*) to 15 (*E. monterogeii*) amastin domain-containing proteins, which is even less than in *Leishmania* (*Mundinia*) spp. [9]. Of note, in all of these proteins, three to four transmembrane domains (TMDs) were identified (Table S12).

**Figure 2:**
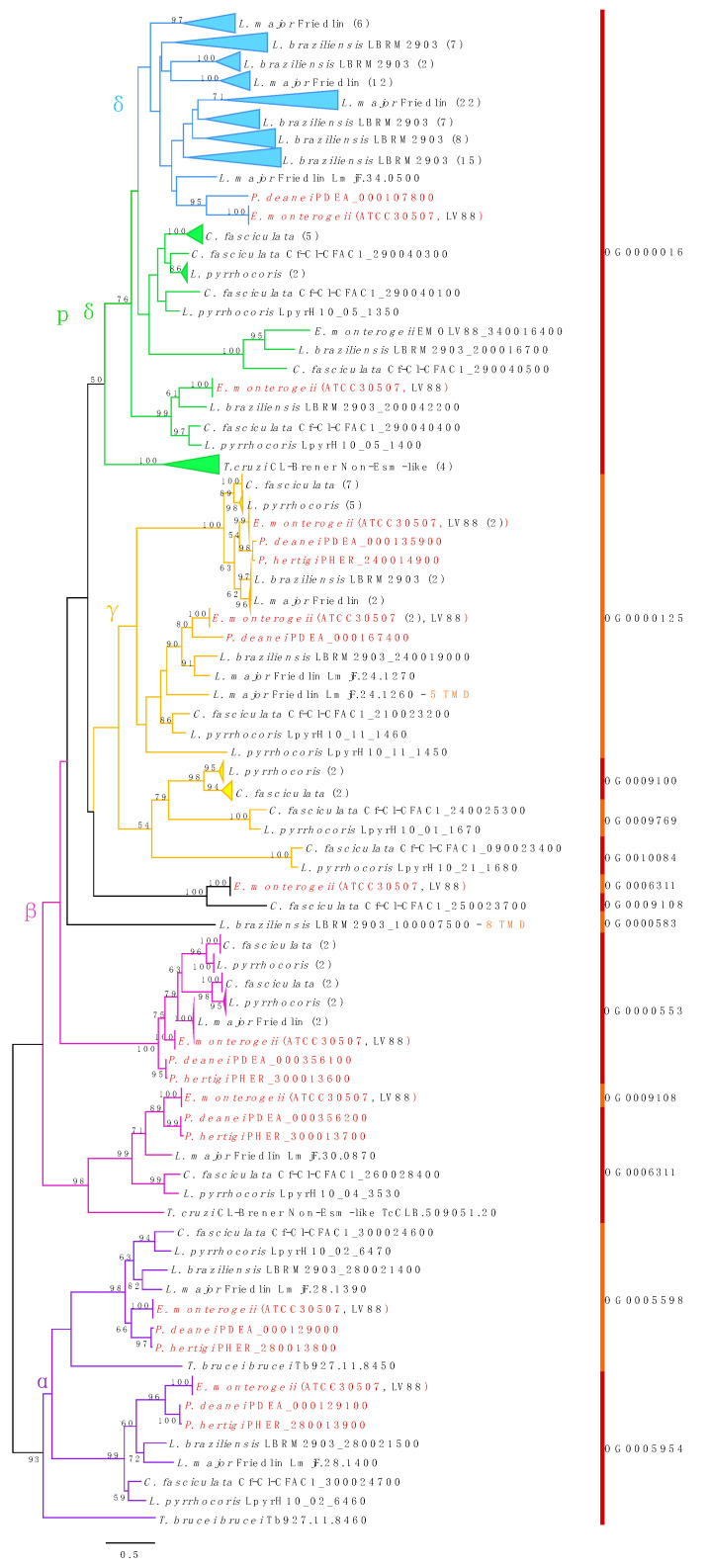
Maximum-likelihood phylogenetic tree of 188 kinetoplastid amastins. Only bootstrap supports over 50% are shown. The sequences obtained in this study are shown in red with the respective OG IDs. The five classes of amastins are highlighted in different colors. Most analyzed proteins have four transmembrane domains (TMDs), with a few exceptions indicated in the tree and Table S12. Numbers of sequences within collapsed clades are shown in brackets.

The amastins repertoire also varies across Leishmaniinae (Figure 2). Based on phylogeny, expression pattern, and secondary structure, these proteins are classified into four subfamilies—α-, β-, γ-, and δ-amastins (including proto-δ-amastins) [85]. While the repertoires of α- and β-amastins are highly conserved across *Leishmania*, *Endotrypanum, Porcisia*, and even monoxenous representatives of the subfamily Leishmaniinae, *P. hertigi* contains a slightly reduced set of γ-amastins, and lacks detectable homologues of proto-δ and δ-amastins (Figure 2). 

The repertoire of δ-amastins is substantially expanded in *Leishmania* as compared to *Endotrypanum* and *Porcisia*. Because some amastin domain-containing proteins that were initially identified by homology-based searches were discarded from phylogenetic analysis based on set threshold (see Materials and Methods for details), we estimated their affinity to known amastin subfamilies by a similarity-based sequence clustering approach using the unfiltered dataset (Figure S6). The composition of the inferred protein clusters strongly corresponds to that of the clades on the amastin phylogenetic tree (Figure 2). Almost all amastin domain-containing proteins of *Endotrypanum*/*Porcisia* that were excluded from the phylogenetic analysis cluster with divergent sequences, which were previously annotated in other Leishmaniinae as putative β-amastins (Figure S6). The exceptions are the two amastin domain-containing proteins of *E. monterogeii* ATCC30507: one is a putative divergent proto-δ amastin (EMON_000317000.1), while, for the other, no affiliation could be established (EMON_000357800.1), similarly to the one of the *P. hertigi* sequences (PHER_000076200.1). 

### 3.9. Biopterin Transporter BT1

The biopterin transporters (BT) are integral membrane proteins [101] of the major facilitator superfamily [102]. The BT1 is a high-affinity biopterin transporter and a low-affinity folate transporter. It is the only non-conjugated pterin transporter and the main biopterin transporter in *Leishmania* [103]. All of the trypanosomatid species with studied metabolism are biopterin auxotrophs [102,104]. Biopterin is a co-factor of endogenous enzymes known to play a role in the parasite’s differentiation and growth. All of the studied *Leishmania* spp. not only possess pterin salvage pathways and pterin transporters, but also the highest number of BT1 family members among trypanosomatids [104]. The BT1 proteins are known to contain 10 to 12 putative TMDs, which are predicted to form amphiphilic α-helixes and β-strands, involved in the formation of aqueous channels across the lipid membrane [105]. All of the kinetoplastid BT1 transporters analyzed here encode six to 24 TMDs, with the majority possessing 12 TMDs (Figure S7, Table S13). Of note, the proteins having similar number of TMDs tend to cluster together, which likely reflecting their shared evolutionary history.

The OG gain/loss analysis showed moderate BT1 repertoire changes in the *Endotrypanum/Porcisia* clade (Table S9). According to the phylogenetic analysis, some BT1 orthologues of *L*. (*Leishmania*) spp. proteins are absent in the *Endotrypanum/Porcisia* spp. (Figure S7). The distribution of the proteins of this family among kinetoplastids suggests that their diversification happened several times during the trypanosomatid evolution (e.g., in the common ancestor of trypanosomes, in that of *Crithidia*/*Leptomonas* clade, *Phytomonas*, and *Crithidia* spp.). In addition, such events also happened in the common ancestor of Leishmaniinae, as judged by the presence of multiple BT1-encoding genes in the representatives of this subfamily and the absence of closely related sequences in trypanosomes. The members of the *Endotrypanum*/*Porcisia* clade apparently secondarily lost some of the BT1 homologues, which are present in other Leishmaniinae (Figure S7). Functional studies are needed to shed light on the role of the reduced BT1 repertoire in *Endotrypanum*/*Porcisia* spp. and whether it plays a role in their pathogenicity.

### 3.10. Notes on Metabolism of Endotrypanum and Porcisia

The metabolic capacities of *Leishmania* spp. have been reviewed elsewhere [76,106,107,108], and they may serve as a reference for the interpretation of the *Endotrypanum/Porcisia* proteomes. The major differences between *L. major* and species under study rest in the absence of many amastin-like genes (discussed above) and hypothetical proteins. Apart from the fact that complete gene families have been missing or reduced to a few gene copies in the *Endotrypanum/Porcisia* clade, the metabolic arsenal of these flagellates is generally similar to that of *L. major*. The main differences include the prominent absence of genes for methionine synthase, methionine synthase reductase, methylmalonyl-CoA epimerase, and methylmalonyl-CoA mutase from the genomes of all three analyzed species. This suggests that *Endotrypanum* and *Porcisia* cannot use the two branched amino acids, Ile and Val, as well as Met, for energy production and gluconeogenesis, because their common degradative intermediate, propionyl-CoA, cannot be converted to succinyl-CoA. On the other hand, the absence of a gene for methionine synthase does not mean that these species are auxotrophic for Met, since all Leishmaniinae possess a second methionine synthase isofunctional enzyme [107]. The enzymes of the methionine salvage pathway are present in all trypanosomatids [76]. The genomes of *Endotrypanum*, *Leishmania*, and *Porcisia* encode a methylthioadenosine phosphorylase that compensates for the loss of a 5-methylthioribose kinase, while monoxenous Leishmaniinae (*C. fasciculata* and *L. pyrrhocoris*) have genes for both of the enzymes. 

Leishmaniinae (*L. major, C. fasciculata, L. pyrrhocoris*) possess two asparaginase isoenzyme genes (orthologues of *LmjF.15.0390* and *LmjF.36.4430*), allowing them to utilize Asn as an energy source. Both isoenzymes usually carry the peroxisomal C-terminal targeting signal (PTS1). Interestingly, the analyzed *Endotrypanum/Porcisia* spp. have lost one of the two genes (orthologue of *LmjF.15.0390*), while the remaining one now also lacks the PTS1.

Within Leishmaniinae, only *C. fasciculata* and *L. pyrrhocoris* have the capacity to transform the bacterial amino acid diaminopimelate into Lys, owing to the acquisition of diaminopimelate decarboxylase and diaminopimelate epimerase genes [89]. The absence of these two genes that were previously reported for *Leishmania* spp. is now also extended for *Endotrypanum/Porcisia* spp. As in all *Leishmania* [109], the gene encoding catalase was not retained in the *Endotrypanum/Porcisia* genomes, further supporting the hypothesis of its incompatibility with the dixenous life cycle [110]. 

No differences between *Leishmania* and *Endotrypanum/Porcisia* with respect to their capacity to synthesize sugar nucleotides were detected, and pools of GDP-Ara, UDP-Fuc, UDP-GlcNAc, GDP-Man, UDP-Glc, UDP-Gal*p*, and UDP-Gal*f* are predicted to be available for the incorporation of the respective sugar residues into glycoproteins on the surface of these flagellates [111]. However, the absence of β-galactofuranosyl/glycosyltransferase in *P. hertigi* and UDP-glucoronosyl and UDP-glucosyl transferase from genomes of all three analyzed species indicates differences in the surface glycoprotein composition between members of this clade and *L. major*.

### 3.11. Selection Analysis

In total, 5901 OGs were found to contain genes from a selected subset of species. Applying a branch-site model A, we identified 280 and 169 genes with positively selected sites (*p*-value below 0.05) on the branches, leading to the *Endotrypanum/Porcisia* and *Leishmania* clades, respectively. Only 19 genes were in the intersection of these two sets. The most commonly found annotations in these lists were ‘hypothetical protein’ and ‘protein containing domain with unknown function’. However, some well-described proteins were also present, e.g., ornithine decarboxylase implicated in the survival of *Leishmania* amastigotes [112] and ascorbate peroxidase, which is essential for the defense against oxidative stress [113]. No considerable GO enrichment for the positively selected genes was documented in the categories ‘biological process’ or ‘molecular function’ on any of the two branches (Figure S8), which indicated that positive selection pressure affects functionally diverse genes. However, the enrichment was revealed in the ‘cellular component’ category for proteins with ‘cilium’ and ‘cell projection’ annotations. These are membrane-bound, intermembrane, and excreted proteins that are commonly involved in host-parasite interactions [114]. 

## 4. Discussion

The genome analysis of *Endotrypanum* and *Porcisia* performed here revealed that these parasites and their morphologically indistinguishable closest phylogenetic relatives, *Leishmania* spp., followed different evolutionary paths, resulting in distinct biology. Although we identified specific sets of genes under positive selection in these two lineages, possibly reflecting their adaptation to different hosts, the number of such genes is rather small. Meanwhile, gene gains and losses, as well as gene family expansions and contractions, show stronger signals in both lineages, indicating that these were the main mode of genome evolution in dixenous Leishmaniinae.

The differences in the metabolic capabilities of *Endotrypanum/Porcisia* spp. and those of *L. major*, as a representative of the genus *Leishmania*, are rather subtle and consist mainly of the repertoire of enzymes participating in the metabolism of amino acids and biosynthesis of surface glycoproteins. More substantial changes can be seen in gene families incorporating hypothetical proteins, about which no definite conclusions can be drawn, as well as in those containing genes encoding members of large gene families, such as membrane proteins, proteins that are involved in cell signaling, parasite–host interaction, and even several families of housekeeping genes (e.g., those encoding motor and DNA repair proteins). Out of these, we identified two protein families, amastins and biopterin transporters BT1, which we find to be at least partially responsible for the differences in pathogenicity between *Endotrypanum/Porcisia* and *Leishmania* spp. based on the drastically different evolutionary patterns of these proteins in the two lineages.

Amastins are transmembrane glycoproteins present on the cell surfaces of all trypanosomatids. In *Leishmania*, with up to ~70 members, the amastins represent the largest developmentally regulated gene family reported so far [100]. These proteins were first identified in *T. cruzi* [115], and they all share similar structural organization with an extracellular domain, several transmembrane segments and an amastin domain. For the majority of amastins, the expression is amastigote-specific and strictly dependent on acidic pH [116,117]. These proteins serve as membrane transporters that are essential for the survival inside the vertebrate cell or as signal transducers allowing for sensing the lysosomal acidic milieu. Amastins are among the most immunogenic leishmanial surface antigens for mice [118] and solicit strong immune responses in humans, which makes these proteins promising vaccine candidates [119]. The amastin repertoire is expanded in *Leishmania* spp. relative to that in other trypanosomatids. The proteins are encoded by a diverse gene family, including four subfamilies (α-, β-, γ-, and δ-amastins), which have distinct genomic positions and diverged already in an ancestral trypanosomatid [85,120]. In *Leishmania* spp., the group of δ-amastins rapidly diversified even further, while such diversification never happened in other trypanosomatids, including the *Endotrypanum/Porcisia* lineage. As a case of extreme reduction, *P. hertigi* lacks detectable homologues of δ-amastins (including divergent sequences of the proto-δ group). Unfortunately, we could not obtain high supports at many important branches of the phylogenetic tree due to a short length of the amastin sequences (most are only 180–210 amino acids long). However, given the amastin distribution patterns that are observed here and in previous studies [84,85,121], we can make certain assumptions regarding the evolutionary history of this protein family in Euglenozoa. The amastin domain-containing proteins with unknown function were already present in the common ancestor of Euglenozoa, as suggested by the presence of the respective homologues in the representatives of all main euglenozoan groups that were analyzed in this respect, except *Perkinsela*, an extremely reduced symbiont of amoebae [84]. As mentioned above, the phylogenetic analysis of amastins incorporating the data from all sequenced genomes is a challenging task, due to the large size of this family and a relatively short protein length. Still, the available data suggest that diversification into α-, β-, γ-, and proto-δ-amastins had not happened later than in the common ancestor of the human pathogenic genera *Leishmania* and *Trypanosoma* (node 42 in Figure 1). The δ-amastin subfamily is apparently *Leishmania*-specific, since no obvious homologues of these sequences were identified with confidence in other trypanosomatids, neither by phylogenetic analysis, nor by using similarity-based protein clustering approach (Figure 2, Figure S6). These genes developed from ancestral proto-δ-amastins and they significantly diversified in the common ancestor of *Leishmania*, while, in *Endotrypanum*/*Porcisia*, they remained scarce [121]. The evolution of the subgenera *L*. (*Leishmania*) and *L*. (*Viannia*) was accompanied by a further diversification of δ-amastins, as judged by the presence of specific clades on the phylogenetic tree of amastins for *L. major* and *L. braziliensis*. In *L*. (*Sauroleishmania*), the repertoire of δ-amastins was secondarily reduced to only two genes [122]. These parasites reside in the bloodstream. Amastigotes (either free or inside monocytes or erythrocytes) are rarely observed and infections are principally detected by culture [123]. To date, there is no evidence that the flagellates seen in the intestine and cloaca of some lizards are *L.* (*Sauroleishmania*) [124]. The view that the expansion of δ-amastin in *Leishmania* was associated with adaptation of the amastigote to the life in vertebrate macrophages [85] is now further supported, since not only *L*. (*Sauroleishmania*), but also *Endotrypanum*/*Porcisia*, which do not infect macrophages, possess a very limited diversity of δ-amastins. Thus, a limited repertoire of δ-amastins in both *L*. (*Sauroleishmania*) and *Endotrypanum*/*Porcisia* is connected to the inability of these pathogens to infect host macrophages. However, while in the ancestor of *Endotrypanum*/*Porcisia*, the δ-amastin family was never expanded, a rather limited set of these proteins in *L*. (*Sauroleishmania*) is likely a result of secondary losses. The results of our phylogenetic analysis are in agreement with the experimental data showing that the knockdown of δ-amastin in *L. braziliensis* affects the parasite-macrophage interaction and results in impaired viability of intracellular amastigotes, which certifies this protein as a virulence factor [125]. Species-specific differences among macrophage-infecting species may be explained by multiple factors, such as the vertebrate host species, the infected macrophage type, which can, in turn, even be affected by the composition of the insect vector’s saliva [126,127]. The phylogenetic distribution of the representatives of other amastin subfamilies suggests that the respective proteins might be functionally significant in the vector, or both vector and host.

The repertoire of biopterin transporters is also narrower in the *Endotrypanum/Porcisia* clade as compared to *L*. (*Leishmania*), but experimental approaches have to address the potential contribution of this feature to the reduced pathogenicity of these parasite. We speculate that, since these proteins are associated with cell differentiation, *Endotrypanum* and *Porcisia* were not forced to develop very precise and diverse mechanisms for this process, as were *Leishmania* spp., which have one of their life cycle stages confined to host macrophages and they demonstrate pronounced antagonistic relationship with the host. While the details of the life cycles and the respective roles of BT1 transporters at its different stages remain to be elucidated for *Endotrypanum* and *Porcisia*, the role of BT1 transporters in several *Leishmania* spp. was clearly connected to survival and growth inside the host macrophages [128]. *L. donovani* cells overexpressing the BT1 gene demonstrated increased infectivity and survival in the macrophages, with the opposite effect being observed in the knock-out cell line [128]. We suggest that, similar to the situation observed for amastins, *Endotrypanum* and *Porcisia* spp. do not require an elaborate repertoire of BT1 transporters, as do macrophage-dwelling *Leishmania*. 

In sum, our genomic analysis of *Endotrypanum* and *Porcisia* spp. allows for a better understanding of the evolutionary trajectories within the dixenous Leishmaniinae and the potentially critical role of the two protein families, amastins and biopterin transporters BT1, in the biology of trypanosomatids. 

## Data Availability

Raw reads and assembled genome sequences were deposited to NCBI database under BioProject accession numbers PRJNA680236, PRJNA680237, and PRJNA680239 for *E. monterogeii* ATCC 30507, *P. deanei* TCC258 and *P. hertigi* TCC260, respectively.

## Supplementary Materials

The following are available online at https://www.mdpi.com/2073-4425/12/3/444/s1, Figure S1: BlobTools statistics for *Endotrypanum monterogeii*. ATCC 30507, *Porcisia deanei* TCC258, and *P. hertigi* TCC260 before and after filtering. Figure S2: Distribution of genomic read coverage for the genome assemblies of *E. monterogeii* ATCC30507 (red), *P. deanei* (blue) and *P. hertigi* (green). Figure S3: The distribution of 50 longest scaffolds of *E. monterogeii* ATCC30507, *P. deanei*, and *P. hertigi* according to the estimated ploidy levels. Figure S4: (A)–(J): Schematic representation of the two-way synteny between the genomes of *Endotrypanum monterogeii* ATCC305007, *Porcisia deanei* TCC258 and *P. hertigi* TCC260 and the reference genome of *L. major* Friedlin. Inverted synteny blocks are in green, direct ones are in red. Only pseudo-chromosome level scaffolds (produced using Companion software) carrying regions of synteny to the respective regions of the reference genomes are shown. (K) The table summarizing the synteny statistics for each pairwise comparison. Figure S5: OGs sharing among kinetoplastids. (A) UpSet plot for the whole dataset of 44 trypanosomatids and the eubodonid *B. saltans* (the species were grouped in accordance with their phylogenomic position, see Table S06 for details). The *Y*-axis represents the intersection size (the number of shared OGs) and the *X*-axis shows the groups/species being compared. The clade *Endotrypanun/Porcisia* is highlighted in pink and the species sequenced in this study are in bold. Red dots indicate OGs uniquely shared among *Endotrypanum* and/or *Porcisia* and unique OGs to each species of this clade. (B) Results of the OG sharing analysis involving only on the *Endotrypanum/Porcisia* group. Figure S6: A sequence similarity network of amastin surface proteins. The network was inferred from a dataset of 237 sequences longer than 100 amino acids using EFI-EST [82] with a BLAST *e*-value threshold of 10^−10^ and a minimum alignment score set to 30. Nodes are color-coded according to the amastin type as follows: putative *α* amastins are in violet, *β* in magenta, proto-δ in green and δ in blue. Sequences not included in phylogenetic analysis due to exclusion criteria (see Materials and Methods), but present in the network, are in pink. When possible, they are putatively annotated based on the phylogenetic analyses (Figure 2 and [121]). Species abbreviations before the protein IDs are as follows: *Crithidia fasciculata* (CFAC1), *E. monteregeii* ATCC30507 (EMON), *E. monteregeii* LV88 (EMOLV88), *Leishmania braziliensis* LBRM2903 (LBRM2903), *L. major* Friedlin (LmjF), *Leptomonas pyrrhocoris* (LpyrH10), *P. deanei* (PDEA)*, P. hertigi* (PHER)*, Trypanosoma brucei brucei* (Tb927) and *T. cruzi* CL-Brener Non-Esmeraldo-like (TcCLB). Figure S7: Maximum-likelihood phylogenetic tree of 320 trypanosomatid BT1 proteins. Only bootstrap supports over 50% are shown. The isolates sequenced and analyzed in this study are shown in red. The numbers of predicted transmembrane domains (TMDs) are indicated beside the protein IDs using different colors (see also Table S13). Figure S8: GO enrichment of positively selected genes. Bar plot showing the GO enrichment of positively selected genes on *Leishmania* (169 genes) and *Endotrypanum/Porcisia* (280 genes) branches. The number of annotated sequences was log-transformed in order to optimize visualization, thus, all the GOs with zero sequence in this plot, contain one sequence. Only GO enrichment with a Fisher *p*-value below 0.05 are shown. Table S1: Statistics of the genome and whole-transcriptome sequencing for *Endotrypanum monterogeii* ATCC 30507 (this work), *Porcisia deanei* TCC258 (this work), *P. hertigi* TCC260 (this work), and *Leishmania major* Friedlin (TriTrypDB). Table S2: Statistics of genome assembly coverage. Table S3: (A) Statistics of cross-mapping of scaffolds of *Endotrypanum monterogeii* ATCC 30507 (this work), *Porcisia deanei* TCC258 (this work), *P. hertigi* TCC260 (this work), and *Leishmania major* Friedlin (TriTrypDB); (B) Statistics of synteny analysis based on the pseudo-chromosome-level scaffolds of *E. monterogeii*, *P. deanei* and *P. hertigi* produced using Companion software. Genome sequence of *L. major* Friedlin was used as a reference. Table S4: Repeated sequences in the analyzed genome assemblies. The upper table contains overall statistics, the bottom one lists identified repeat families. Table S5: Dataset used in the phylogenomic analysis. Species, whose genomes and transcriptomes were sequenced in this work, are in bold. Table S6: Grouping criteria used for OG sharing analysis. Table S7: Functional annotation of proteins belonging to OGs shared among various trypanosomatids groups. Table S8: Counts for gene family gains/losses/expansions/contractions for the branches and nodes of the phylogenomic tree. Table S9: Gene family gains and losses for the species under study. Table S10: Gene family expansions and contractions for the species under study. Table S11: Pairwise identity matrix of 239 amastin domain-containing proteins in trypanosomatids. Sequences removed from further analysis (see Materials and Methods for exclusion thresholds) are highlighted in red. Table S12: Composition of collapsed clades in the amastin phylogenetic tree. The sequences are listed in the same order as in the tree. Table S13: Composition of collapsed clades on the BT1 tree. The sequences are listed in the same order as in the tree. The lower table contains a list of species abbreviation used on the BT1 tree.
