# Supplementary material for: Genome Analysis of Endotrypanum and Porcisia spp., Closest Phylogenetic Relatives of Leishmania, Highlights the Role of Amastins in Shaping Pathogenicity
Source: Genes (Basel). 2021 Mar 20;12(3):444. doi: 10.3390/genes12030444 (PMC8004069; doi:10.3390/genes12030444)
Supplement: Supplementary file 1 [file genes-12-00444-s001.zip › Figure S1.pdf]

Endotrypanum monterogeii ATCC30507

Before decontamination

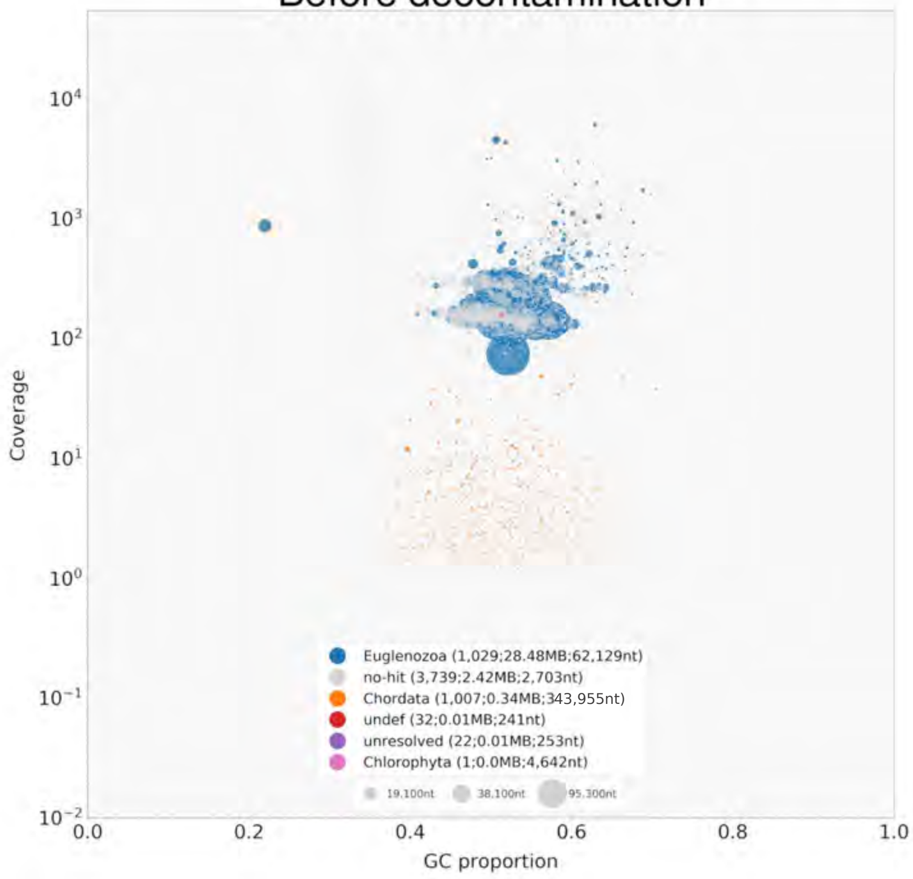

Unmapped: 0.28%; Mapped: 99.72%

After decontamination

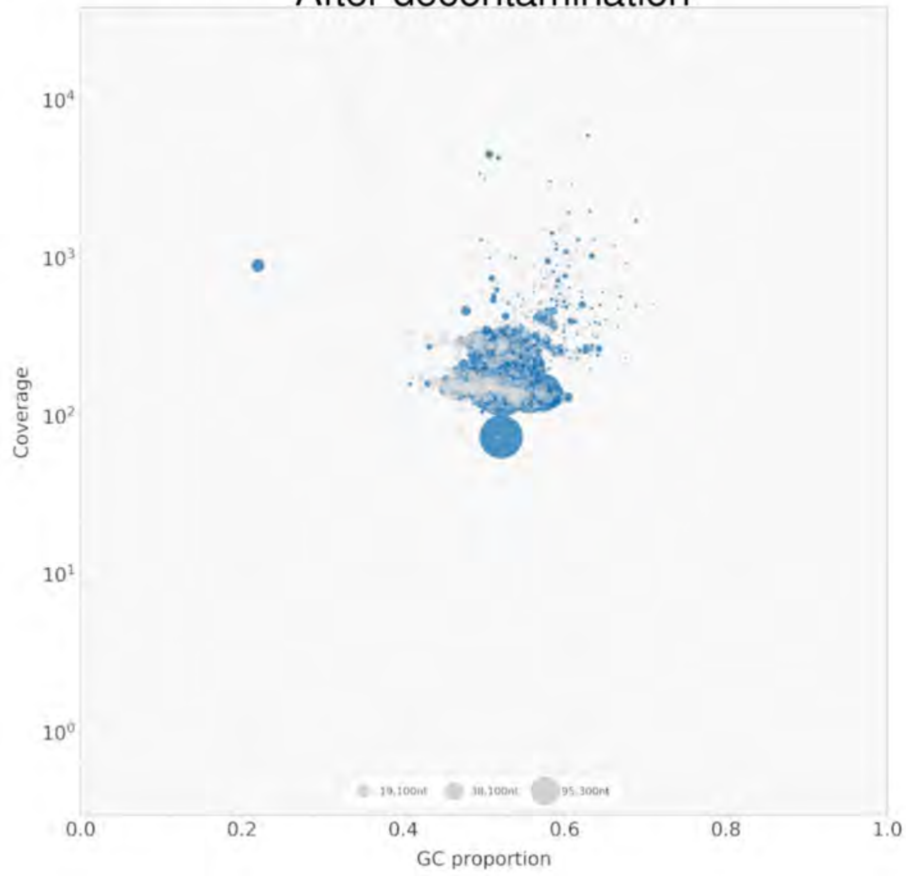

Unmapped: 0%; Mapped: 100% (Euglenozoa: 80.7%, no-hit: 19.3%,

Porcisia deanei TCC258

Before decontamination

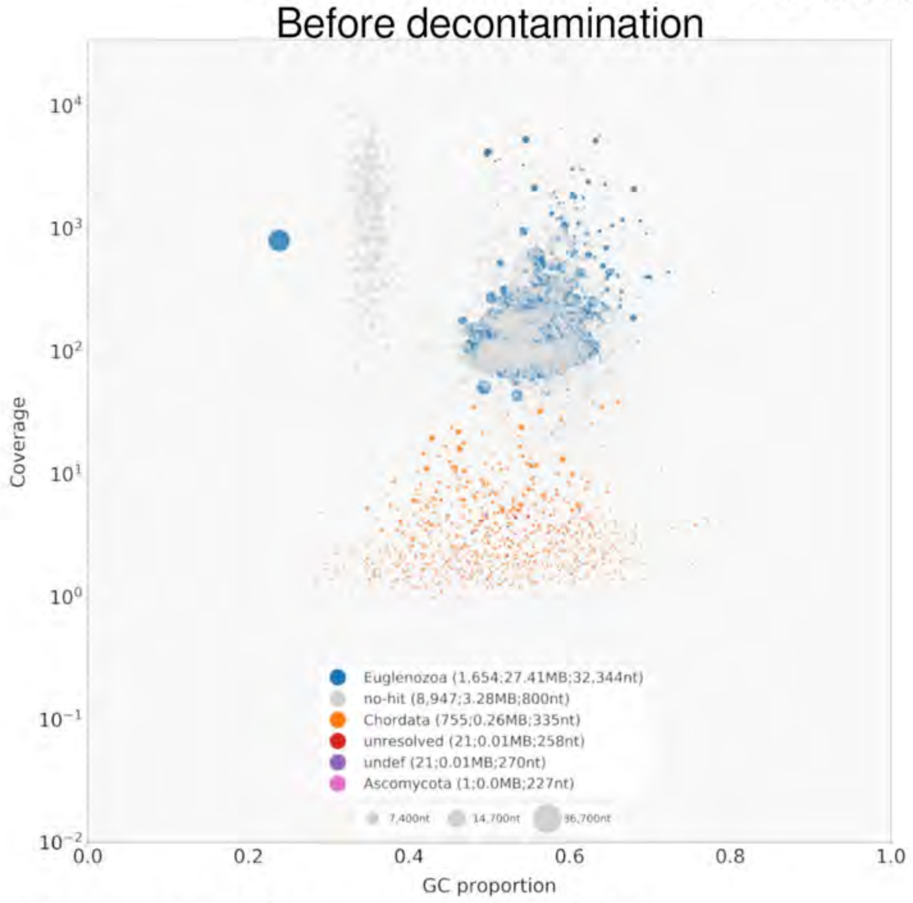

Unmapped: 0.69%; Mapped: 99.31%

After decontamination

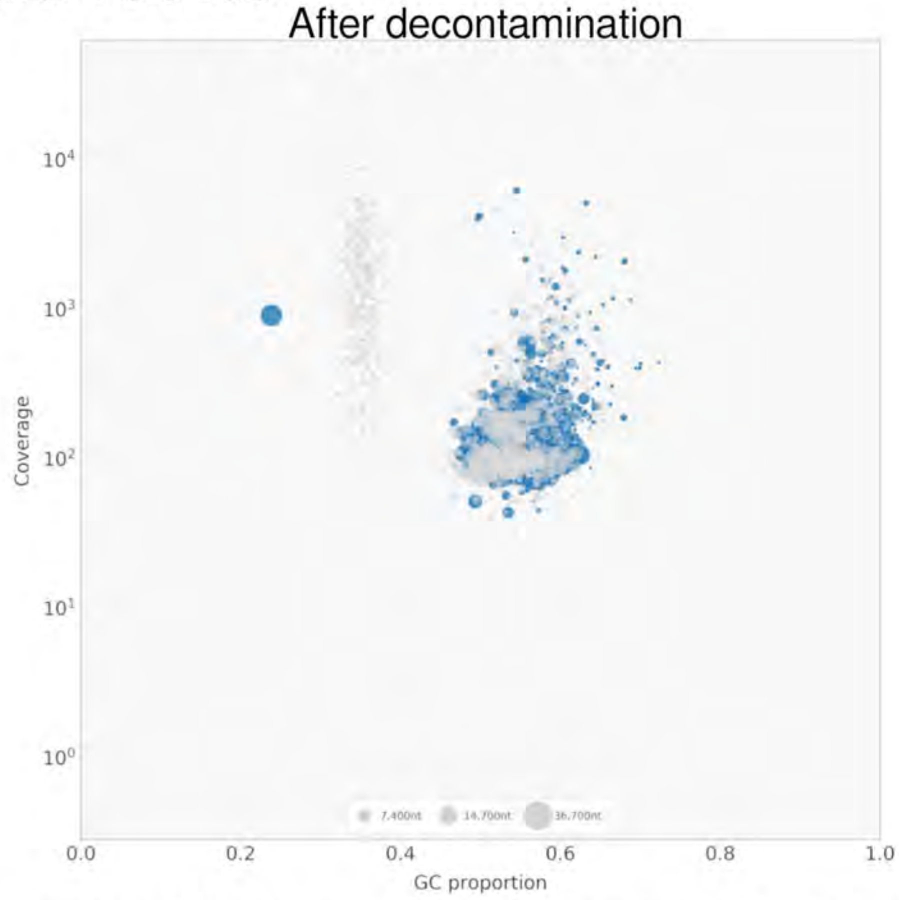

Unmapped: 0%; Mapped: 100% (Euglenozoa: 73.67%, no-hit: 26.33%,

Porcisia hertigi TCC260

Before decontamination

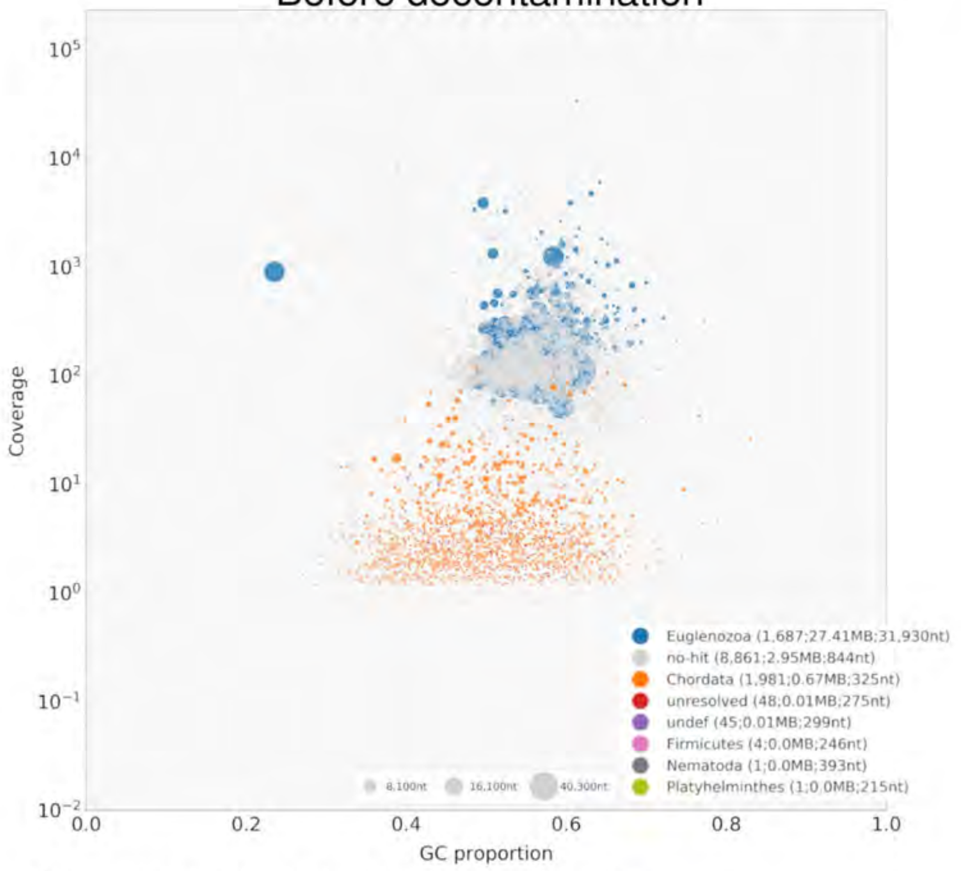

Unmapped: 2.53%; Mapped: 97.47%

After decontamination

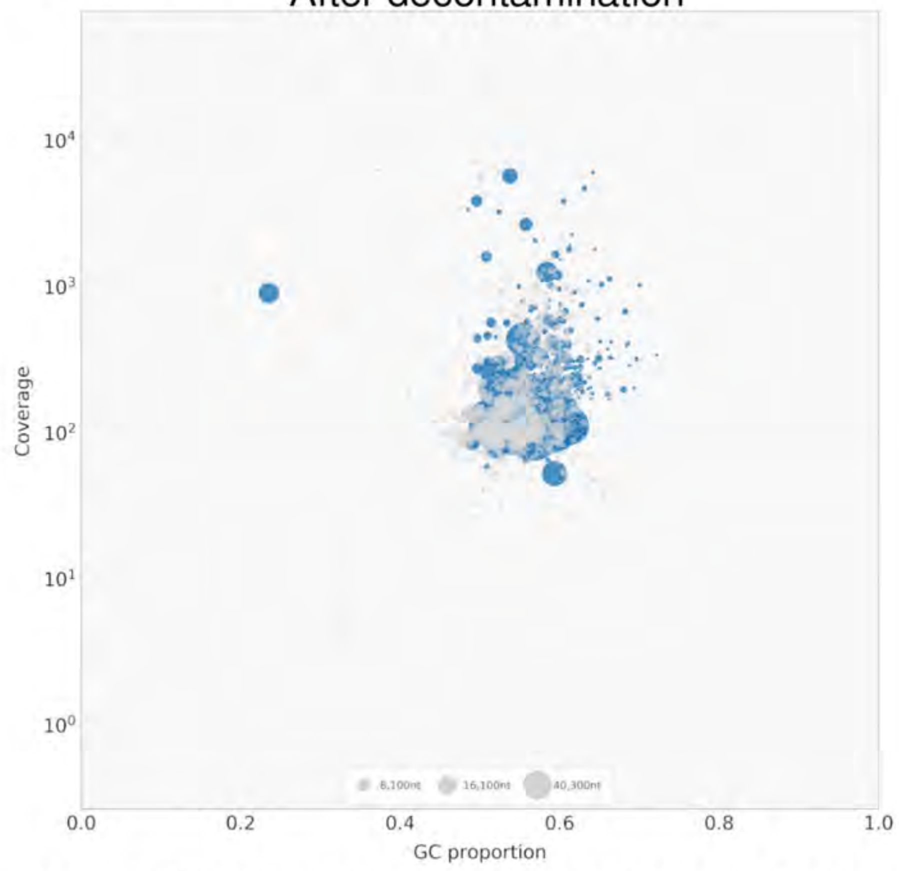

Unmapped: 0%; Mapped: 100% (Euglenozoa: 84.31%, no-hit: 15.69%
