# Supplementary material for: Genome Analysis of Endotrypanum and Porcisia spp., Closest Phylogenetic Relatives of Leishmania, Highlights the Role of Amastins in Shaping Pathogenicity
Source: Genes (Basel). 2021 Mar 20;12(3):444. doi: 10.3390/genes12030444 (PMC8004069; doi:10.3390/genes12030444)
Supplement: Supplementary file 1 [file genes-12-00444-s001.zip › Figure S4.pdf]

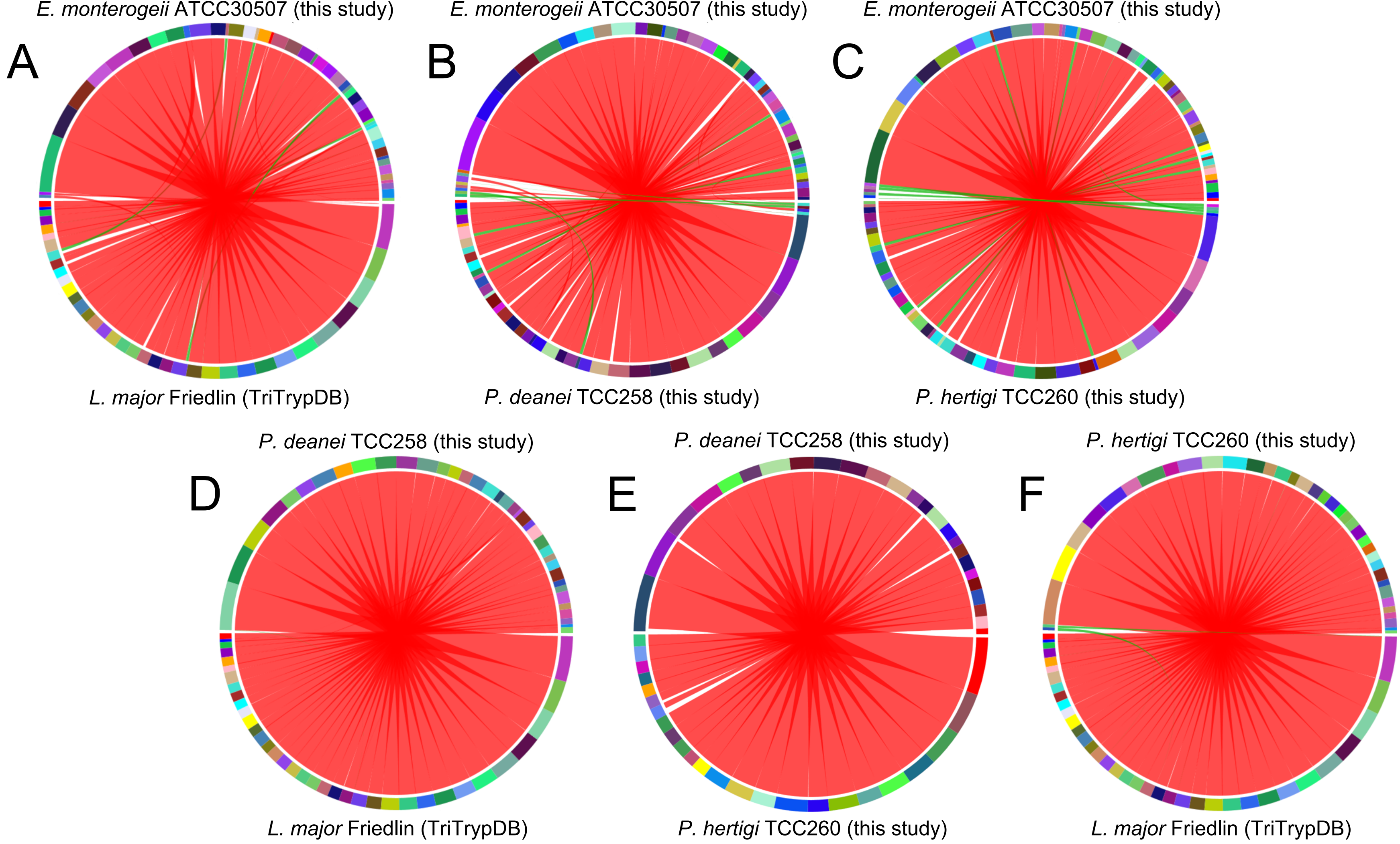

	Species	Chromosomes	Total kb	# genes	% of sequence covered by genes	# of anchors	% of anchors in blocks	% of anchors annotated	% of coverage by anchors	# of blocks	% double coverage	# of inverted blocks	% of genes within synteny blocks
**A**	*Endotrypanum monterogeii* ATCC30507	35	30,584	7,695	46	7,186	55	94	46	155	0	53	82
	Leishmania major Friedlin	36	32,855	10,354	50	7,186	55	97	42	155	0	53	50
**B**	*Endotrypanum monterogeii* ATCC30507	35	30,584	7,695	46	7,768	35	92	51	113	0	39	56
	Porcisia deanei TCC258	37	29,784	7,802	48	7,768	35	94	52	113	0	39	47
**C**	*Endotrypanum monterogeii* ATCC30507	35	30,584	7,695	46	7,761	34	93	51	114	0	37	54
	Porcisia hertigi TCC260	37	29,350	7,818	49	7,761	34	94	53	114	0	37	50
**D**	*Porcisia deanei* TCC258	37	29,784	7,802	48	7,580	52	96	49	68	0	20	84
	Leishmania major Friedlin	36	32,855	10,354	50	7,580	52	97	46	68	0	20	41
**E**	*Porcisia deanei* TCC258	37	29,784	7,802	48	8,425	55	71	72	37	0	0	46
	Porcisia hertigi TCC260	37	29,350	7,818	49	8,425	55	71	73	37	0	0	46
**F**	*Porcisia hertigi* TCC260	37	29,350	7,818	49	7,565	52	96	50	71	0	16	85
	Leishmania major Friedlin	36	32,855	10,354	50	7,565	52	98	46	71	0	16	42
