# Supplementary figures and images for: Genome Analysis of Endotrypanum and Porcisia spp., Closest Phylogenetic Relatives of Leishmania, Highlights the Role of Amastins in Shaping Pathogenicity
Source: Genes (Basel). 2021 Mar 20;12(3):444. doi: 10.3390/genes12030444 (PMC8004069; doi:10.3390/genes12030444)

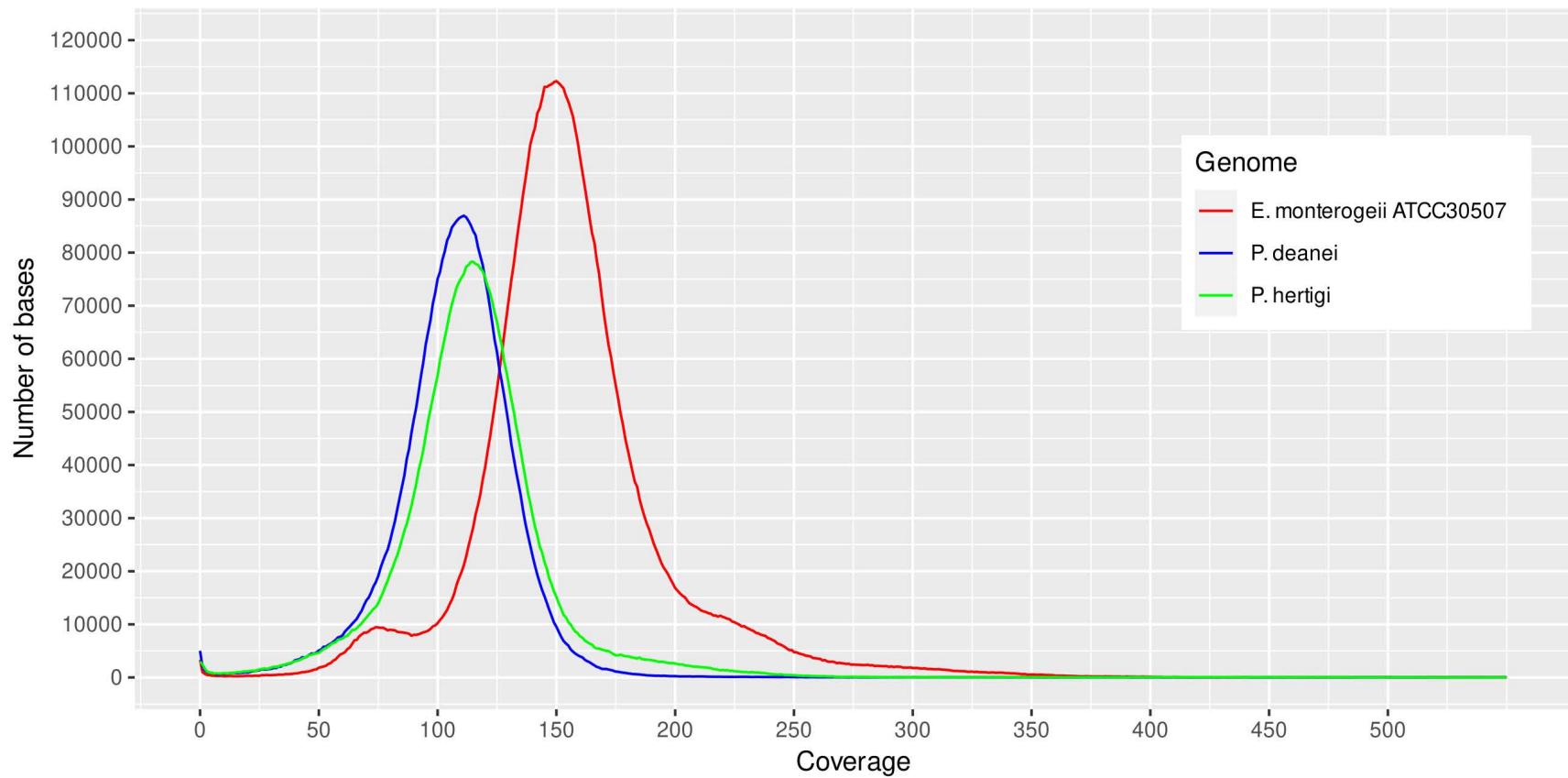

Supplement: Supplementary file 1 [file genes-12-00444-s001.zip › Figure S2.pdf]

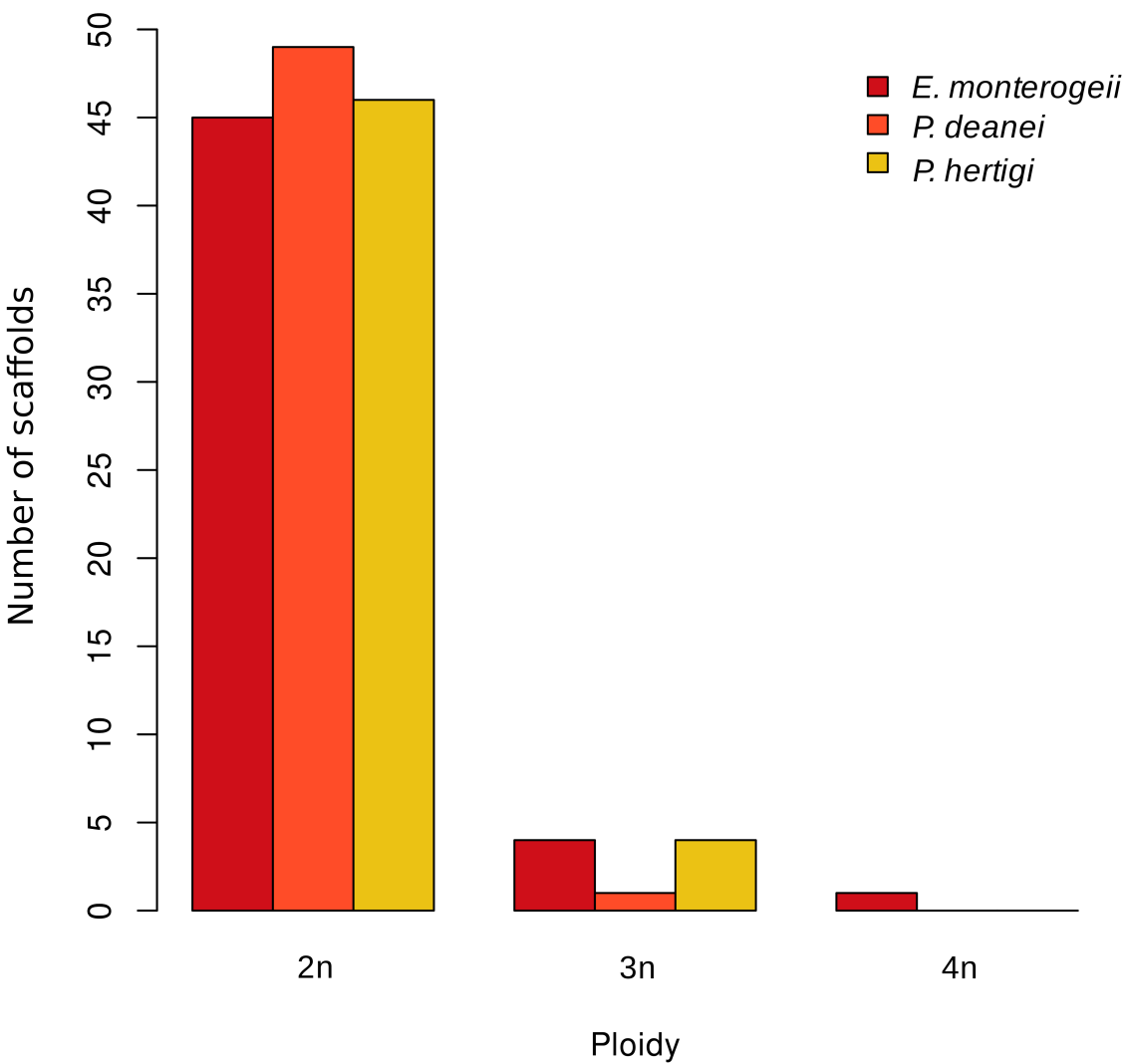

Supplement: Supplementary file 1 [file genes-12-00444-s001.zip › Figure S3.pdf]

A

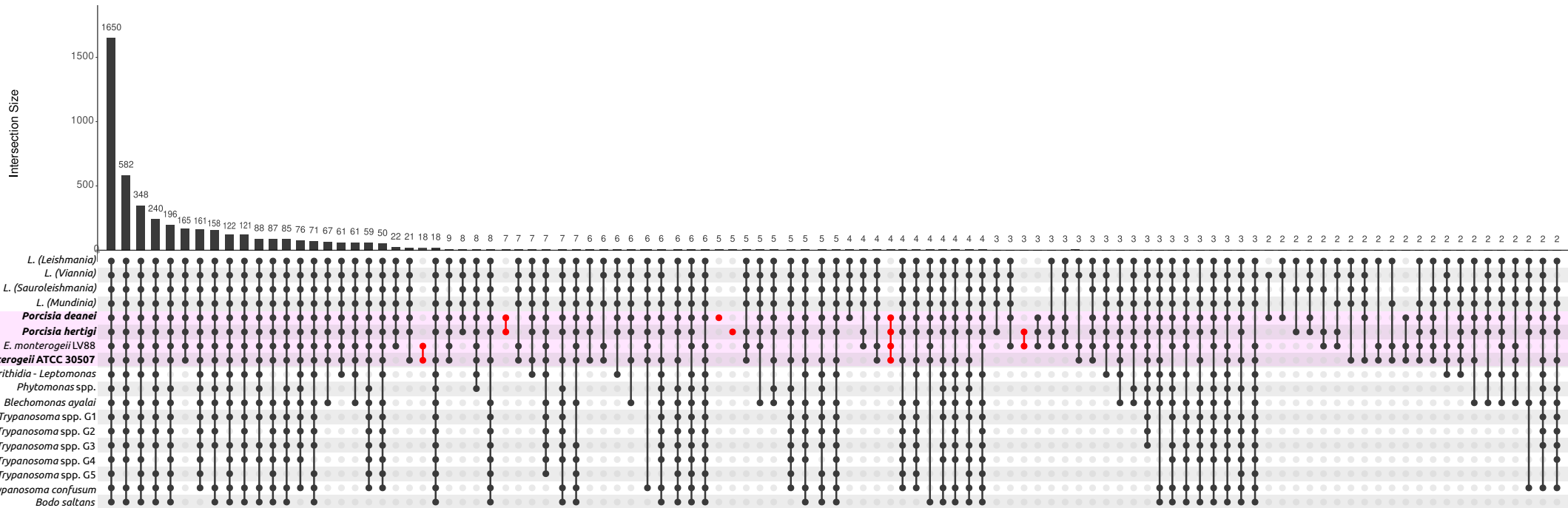

B

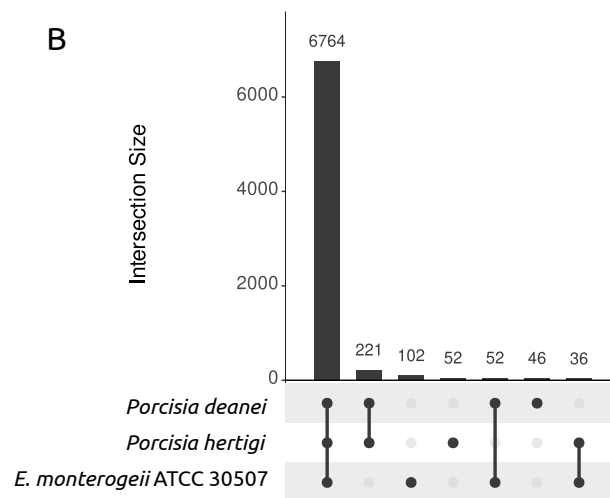

Supplement: Supplementary file 1 [file genes-12-00444-s001.zip › Figure S5.pdf]

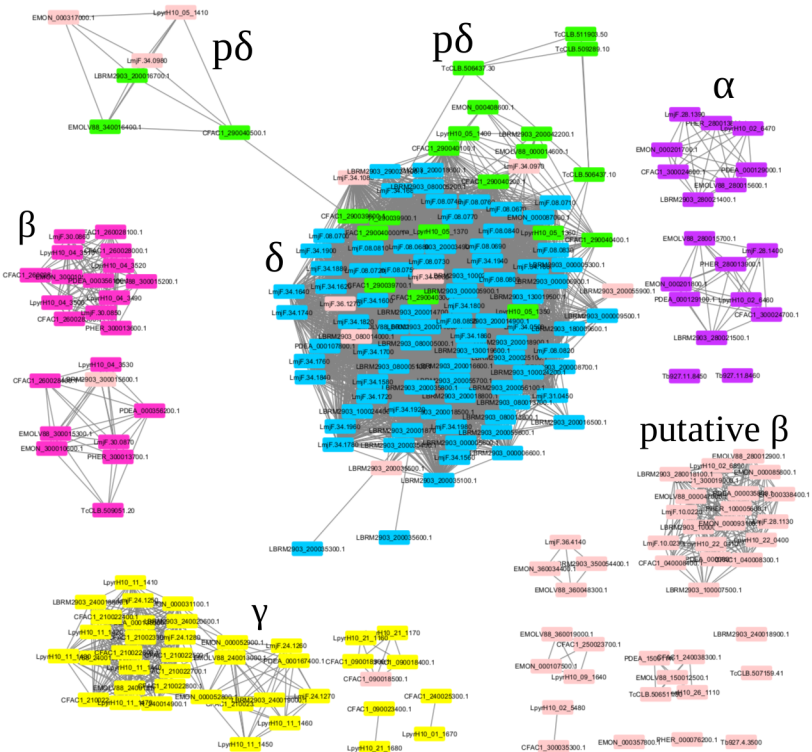

Supplement: Supplementary file 1 [file genes-12-00444-s001.zip › Figure S6.pdf]

# Leishmania major

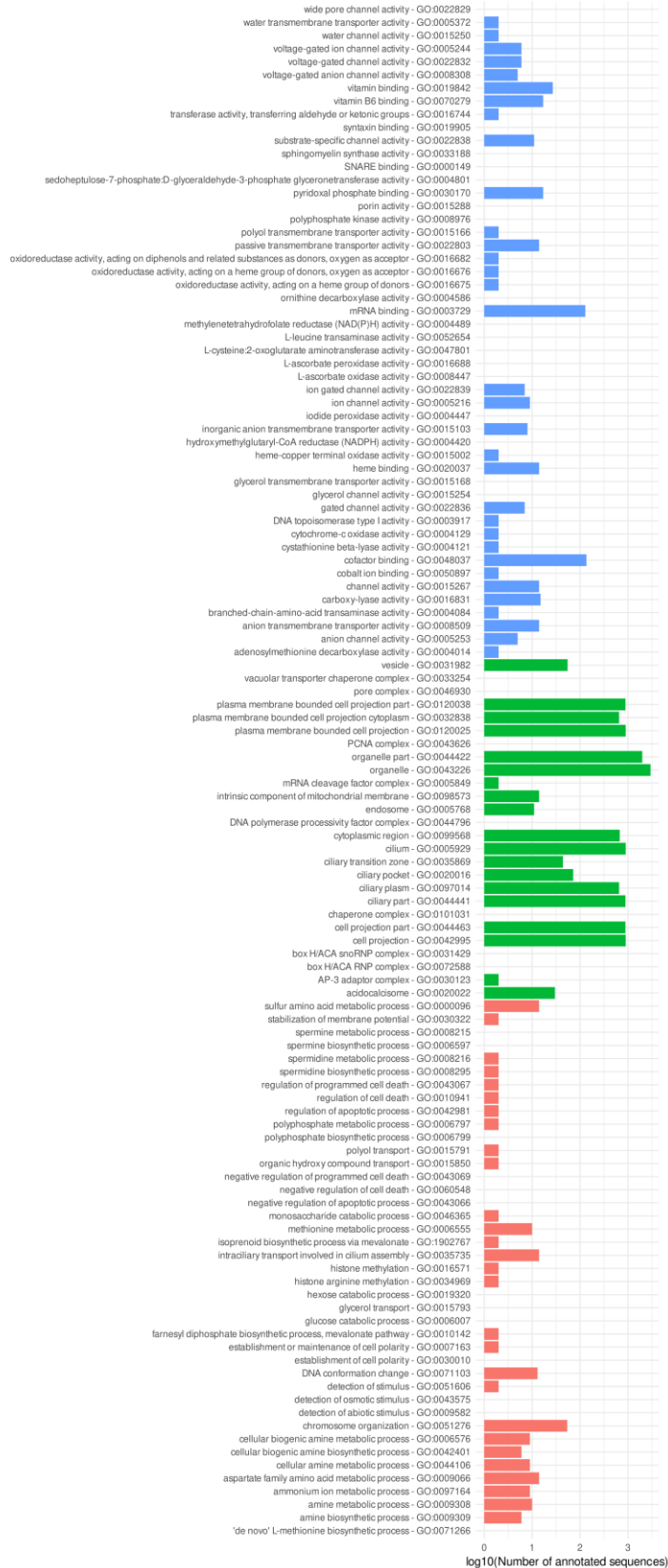

# Endotrypanum-Porcisia

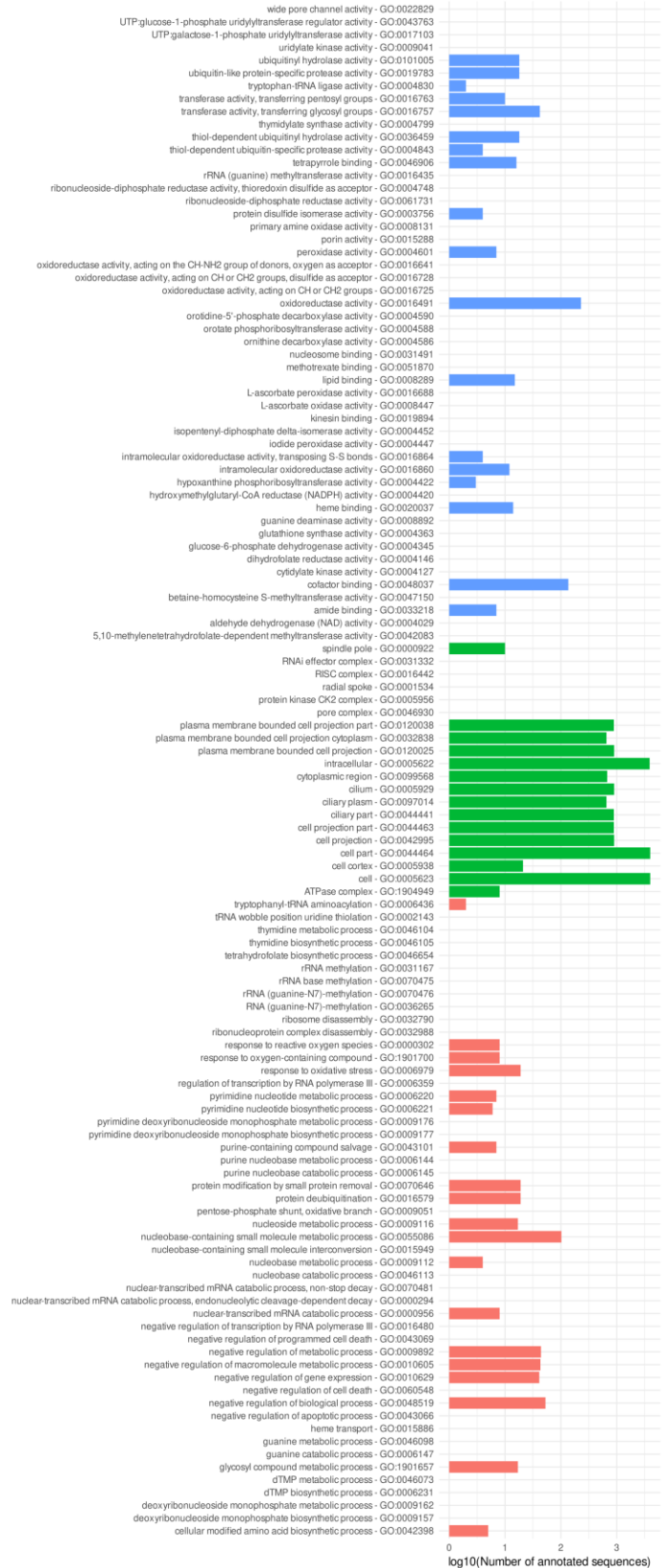

Supplement: Supplementary file 1 [file genes-12-00444-s001.zip › Figure S8.pdf]
