# Supplementary material for: Genome Analysis of Endotrypanum and Porcisia spp., Closest Phylogenetic Relatives of Leishmania, Highlights the Role of Amastins in Shaping Pathogenicity
Source: Genes (Basel). 2021 Mar 20;12(3):444. doi: 10.3390/genes12030444 (PMC8004069; doi:10.3390/genes12030444)
Supplement: Supplementary file 1 [file Figure S7.pdf › Figure S7.pdf]

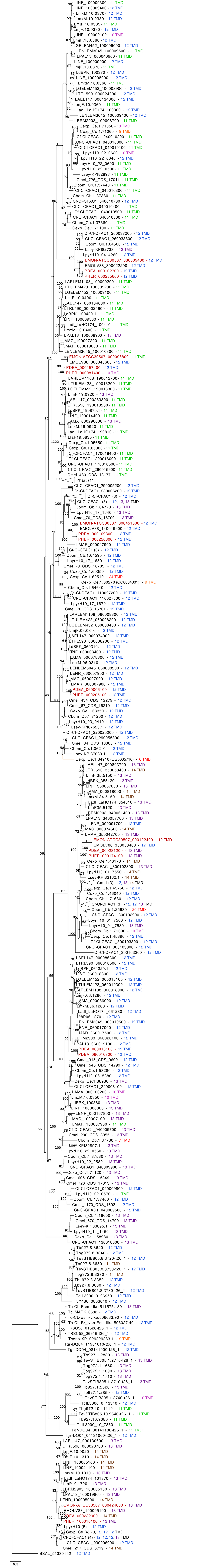
BSAL_51330-t42 - 12 TMD
0.5
